# Advancing chronic and acute wound healing with cold atmospheric plasma: cellular and molecular mechanisms, benefits, risks, and future directions

**DOI:** 10.3389/fmed.2025.1527736

**Published:** 2025-02-26

**Authors:** Nastaran Raissi-Dehkordi, Negar Raissi-Dehkordi, Hamed Ebrahimibagha, Tahereh Tayebi, Kasra Moeinabadi-Bidgoli, Mohammad Hassani, Hassan Niknejad

**Affiliations:** ^1^Department of Pharmacology, School of Medicine, Shahid Beheshti University of Medical Sciences, Tehran, Iran; ^2^Department of Vascular and Endovascular Surgery, Taleghani General Hospital, Tehran, Iran

**Keywords:** cold atmospheric plasma, wound healing, molecular mechanisms, angiogenesis, inflammation, antimicrobial, infection, translational medicine

## Abstract

Chronic and acute wounds represent significant challenges in healthcare, often leading to prolonged recovery times and increased complications. While chronic wounds, such as diabetic foot ulcers and venous leg ulcers, persist due to underlying conditions and biofilm formation, acute wounds, including surgical incisions and burns, can also benefit from innovative therapeutic approaches. Cold atmospheric plasma (CAP) has emerged as a promising non-invasive therapy capable of enhancing wound healing outcomes across both wound types. This review examines the cellular and molecular mechanisms by which CAP promotes wound repair, focusing on its modulation of inflammation, stimulation of angiogenesis, facilitation of tissue remodeling, and antimicrobial effects, which can potentially be used in regenerative medicine. CAP generates reactive oxygen and nitrogen species that influence key cellular processes, accelerating tissue regeneration while reducing bacterial load and preventing biofilm formation. Clinical applications of CAP have demonstrated its efficacy in improving wound healing metrics for both chronic and acute wounds. Despite promising results, translating CAP into routine clinical practice requires addressing challenges such as standardizing treatment protocols, assessing long-term safety, and developing portable devices. Future research should prioritize optimizing CAP parameters and exploring combination therapies to maximize its therapeutic potential. Overall, CAP represents a safe, effective, and versatile modality in wound management, with the potential to significantly improve patient outcomes in both chronic and acute wound care.

## Graphical abstract

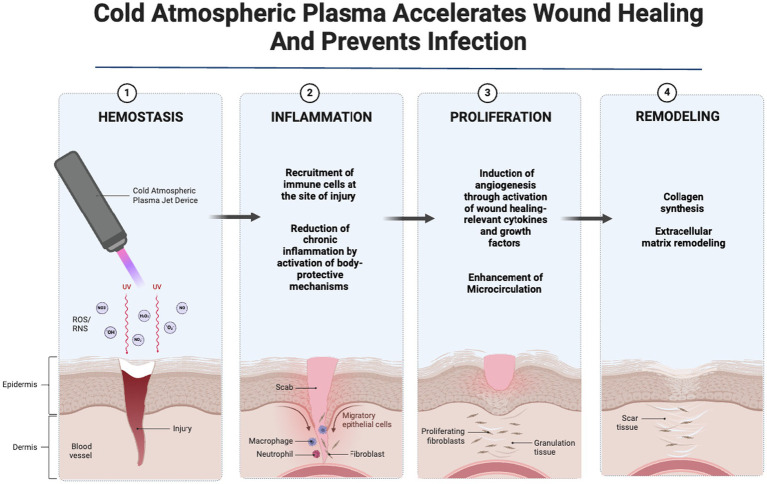

## Introduction

Chronic and acute wounds pose a significant burden to global healthcare systems due to their impact on patient outcomes and resource utilization (1). Successful wound healing progresses through the interconnected phases of hemostasis, inflammation, proliferation, and remodeling (2). Acute wounds such as surgical wounds and burns usually heal efficiently by progressing through the typical phases of wound healing, while chronic wounds such as diabetic foot ulcers and pressure ulcers initiate the healing process but experience extended stages of inflammation, proliferation, or remodeling, resulting in fibrotic tissue and development delayed healing (3).

Various categories of chronic wounds are identified, including venous and arterial insufficiency ulcers, diabetic foot ulcers, and pressure ulcers (4). Chronic wounds present a major and complex challenge to global health, leading to higher rates of morbidity and mortality worldwide. Approximately 10.5 million people, predominantly patients with diabetes and the elderly, are affected by chronic wounds in the United States (5), a number expected to grow due to an aging population (6). Chronic wounds lead to prolonged hospitalization, compromised quality of life, increased risk of systemic infection, amputation, and mortality, imposing high costs to the global health system (7).

Underlying conditions such as diabetes, venous and arterial insufficiency, neuropathies, or prolonged immobility leading to pressure ulcers are responsible for the pathogenesis of chronic wounds (8). Polymicrobial wound infection and the ensuing pathologic inflammation, together with biofilm production, contribute to chronicity of wounds (9). Skin injury disrupts the skin barrier, leading to microbial contamination followed by proliferation and colonization of microorganisms in the wound area, which later develop biofilms in chronic wounds (10). Biofilms comprise various bacterial species which are closed off in a protective glycocalyx and attached to the wound surface (11). Contamination source may be from the normal microbiota from the surrounding skin, exogenous, or endogenous microbiome (12).

Current wound care methods are expensive, time-consuming, and only partially effective, underscoring the need for innovative treatment approaches (13, 14). Cold atmospheric plasma (CAP) is a potential option for acute and chronic wound healing due to its unique biological properties (15). Cold plasma is a partially ionized gas generated from plasma exposed to a high-strength electrical field at atmospheric pressure and room temperature (16), resulting in the production of a mix of reactive oxygen and nitrogen species (ROS and NOS, respectively), UV radiation, heat, and ions (17). Various gas sources, such as helium, argon, nitrogen, and air, can be utilized to generate CAP (18). Adjusting the gas composition influences the types of reactive species generated and can be customized for specific purposes such as bacterial inactivation (19).

## Generation methods (dielectric barrier discharge and atmospheric pressure plasma jet)

**Figure 1:**
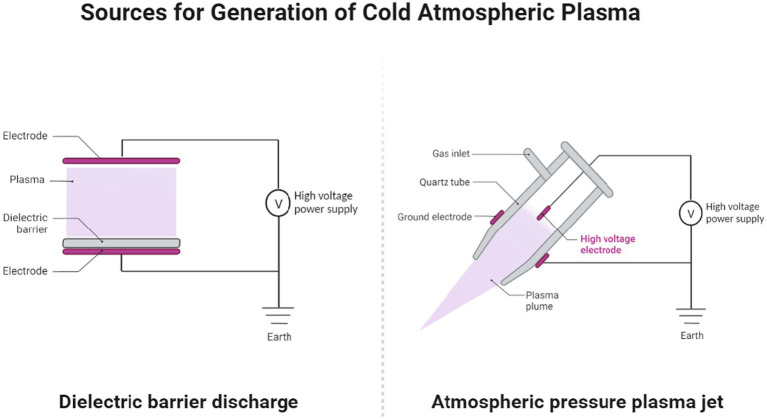
The two primary sources for cold atmospheric pressure plasma (CAP) generation: dielectric barrier discharge (DBD) and atmospheric pressure plasma jet (APPJ). Created with Biorender.com.

The two commonly used sources for generating CAP are dielectric barrier discharge (DBD) and atmospheric pressure plasma jet (APPJ), each with unique characteristics and operating principles (Figure 1). Direct sources such as DBD devices utilize a dielectric-protected powered electrode and a secondary electrode (the human or animal body, such as the skin or organ) to create stable plasma and are popular for low-temperature plasma applications, providing stable discharge without electric arcs. Adherence to safety standards is essential for DBD devices to ensure nondestructive treatment. In contrast, APPJ systems, as indirect plasma sources, utilize the afterglow of plasma or plasma-activated liquid to direct a high-frequency power supply to a small nozzle in order to produce a focused plasma plume and are widely used for treating heat-sensitive materials and operating in ambient air, making them a versatile choice across various applications (20–22). The choice between these plasma sources depends on the specific application, desired plasma characteristics, and target area for treatment. While DBD devices are suitable for larger wound areas with diffuse plasma, plasma jets offer the precision needed for localized treatments. Other less-commonly used sources for production of plasma include microwave plasma systems, microhollow cathode systems, plasma arrays, and on-chip plasmas (23).

Plasma-based wound care promotes healing by generating ROS/RNS, influencing cell signaling, and increasing cytokines and growth factors, which drive angiogenesis, proliferation, and inflammation resolution. Understanding plasma-induced cellular changes is key for clinical use. This review explores the therapeutic potential of CAP for wound healing, focusing on its molecular mechanisms, antimicrobial effects, and clinical applications. We discuss how CAP modulates key biological pathways involved in wound healing, including inflammation, angiogenesis, and tissue remodeling. CAP’s antimicrobial properties are reviewed, including its ability to reduce microbial load and prevent biofilm formation in chronic wounds. Finally, the review addresses the current clinical evidence supporting the use of CAP in wound care and outlines future directions for its integration into clinical practice.

## Mechanisms of cold atmospheric plasma action in chronic wound healing

### Effects on inflammation

The role of inflammation in chronic wound healing involves intricate interactions among various cellular components and molecular mediators (24). Cold plasma therapy is a promising approach to modulate inflammation and optimizing wound healing outcomes. When applied to the wound, non-thermal plasma interacts with the inflammatory cells and molecular mediators involved in the healing process and has been shown to recruit immune cells at the site of injury, while preventing chronic inflammation through promoting the release of anti-inflammatory factors. This section examines how CAP influences inflammation and the underlying molecular pathways, with a focus on H2O2, peroxisome proliferator-activated receptors (PPARs), and nuclear factor erythroid 2-related factor 2 (NRF2). A deeper understanding of these mechanisms may pave the way for new strategies to manage inflammation and improve chronic wound healing outcomes.

### Effects on immune cell recruitment in chronic wound healing

Reducing inflammation is an integral part of chronic wound healing, characterized by complex interactions among resident cells, infiltrating leukocytes, extracellular matrix (ECM) molecules, and various modulators (25). Tissue injury activates chemokines and cytokines, recruiting immune cells and activating macrophages (26). Exposure of human dermal fibroblasts to plasma therapy results in upregulation of inflammatory cytokines (IL-6 and IL-8), activation of MCP-1, and increased levels of GRO *α*, a chemokine known for its chemo-attractant activity in inflammation (27). Induction of these molecules by CAP creates a pro-inflammatory microenvironment at the wound site, promoting the influx of immune cells crucial for effective wound healing. Furthermore, regulators of tissue remodeling, TGF-ß1/2, and Serpine E1 (PAI-1), exhibit enhanced expression following cold plasma exposure. Notably, the induction of CD154 (CD40 ligand) indicates a potential contribution to leukocyte recruitment and immune responses (27). Cold plasma generates hydrogen peroxide (H2O2), which activates macrophages and triggers the inflammatory response needed for healing (28). As a signaling molecule, H2O2 modulates key transcription factors such as NF-κB, promotes the secretion of inflammatory cytokines and attracts leukocytes to the wound site (29, 30). The choice of gas in plasma generation influences the chemical behavior of H2O2 and its biological effects. In a recent study, researchers explored how various working gases (argon, helium, and air) used in plasma generation affect the production of hydrogen peroxide and its subsequent impact on mammalian tissue. While helium-based plasma effectively produces active H2O2, switching to other gasses results in the inactivation of H2O2. This inactivation might be attributed to the interaction of H2O2 with chloride and reactive nitrogen species (RNS), which are more prevalent in environments using air or argon than in those using helium (31). Recognizing the gas-dependent effects on H2O2 activity helps tailor cold plasma treatments for chronic wounds, such as diabetic ulcers and burns, to better control inflammation and infection.

Generation of ROS and RNS by cold plasma is pivotal in its effects, as it can modulate various cellular processes, including the activity of PPAR-*γ* (32). PPARs are a group of nuclear receptors that regulate various cellular processes, including inflammation and metabolism. Cold plasma exposure increases ROS levels in fibroblast-like cells, likely by oxidizing sulfide or lipid groups.

This increase in ROS levels subsequently upregulates PPAR-γ expression in the primary cells. Consequently, there is an increase in the generation and release of IL-6, a cytokine crucial for tissue regeneration, signaling immune cells to the injury site. Interestingly, while IL-6 levels are notably elevated following non-thermal plasma exposure, whereas other cytokines such as IL-1b, TNF-*α*, or TGF-beta show no notable changes (32). By enhancing IL-6 secretion, PPAR-γ activation following therapeutic plasma exposure may attract neutrophils and macrophages to the injury site, facilitating the inflammatory phase essential for initiating the wound healing process.

### Effects on reduction of chronic inflammation in chronic wounds

**Figure 2:**
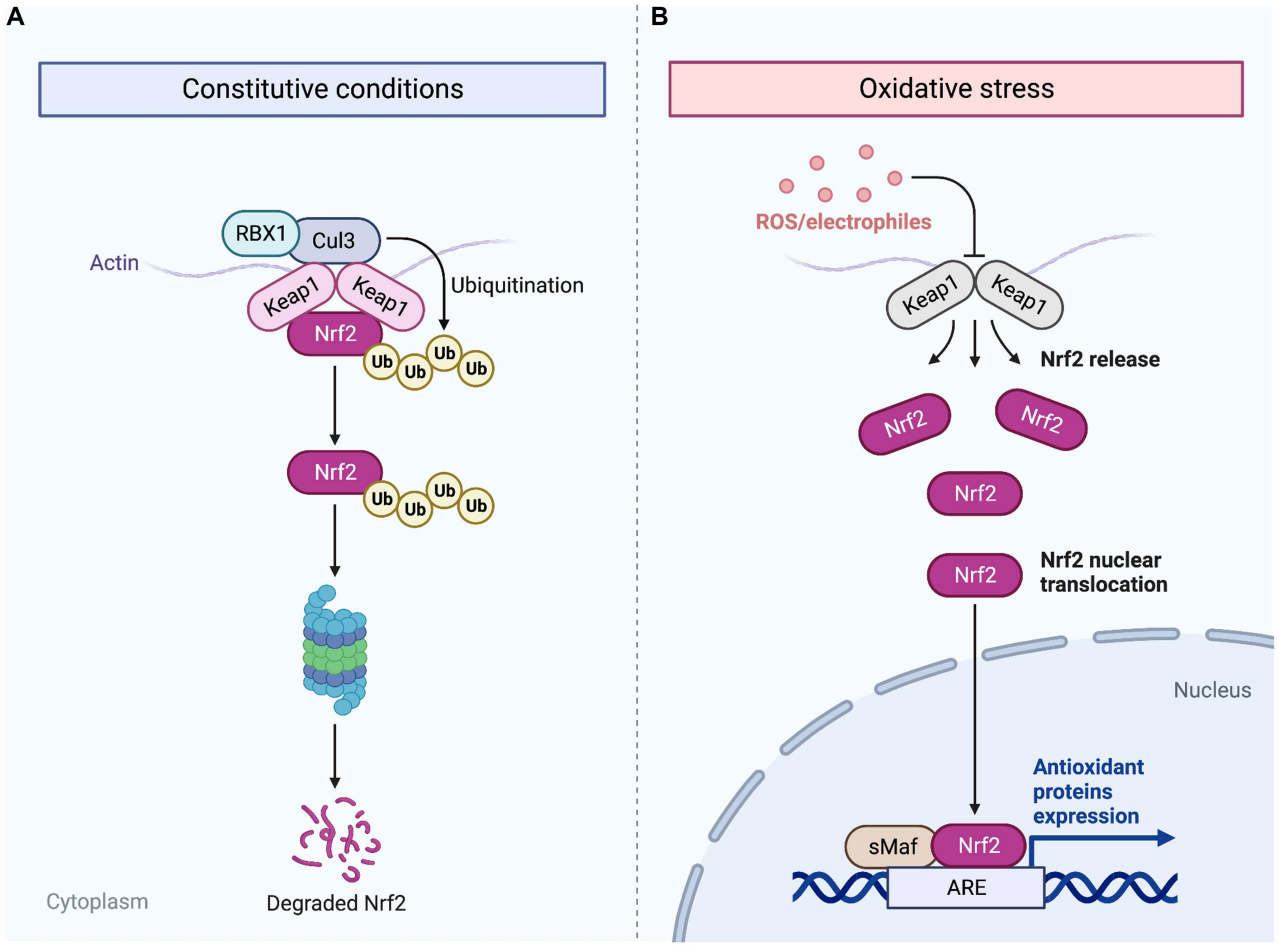
Mechanism of NRF2 activation by ROS generated from CAP. Following cold plasma therapy, ROS are generated, which indirectly lead to the aggregation of NRF2. Normally, under homeostatic conditions, KEAP1 binds to NRF2 in the cytoplasm, facilitating its ubiquitination and subsequent proteasomal degradation **(A)**. However, the ROS produced by plasma jet exposure inactivate KEAP1, thus preventing the degradation of NRF2 **(B)**. Cold plasma exposure allows NRF2 to accumulate and migrate to the nucleus where it binds to the antioxidant response element (ARE) on DNA. This interaction enhances the transcription of antioxidant genes, initiating a robust antioxidant defense response following treatment with CAP. Created with Biorender.com.

Chronic inflammation, characterized by prolonged and dysregulated immune response, can hinder the progression of wound healing and lead to impaired tissue regeneration. Cold plasma therapy aids in management of chronic inflammation in wounds by activating key detoxifying enzymes and reducing oxidative stress in the affected tissues. As shown in Figure 2, CAP initiates a series of antioxidant and detoxification enzymes by promoting the expression of NRF2 (33). Over the past thirty years, NRF2 has gained significant attention for its crucial antioxidant role in maintaining cellular redox balance, making it a potential target for chronic inflammation control in wound healing. In human diabetic ulcers, NRF2 activation in the epidermis suggests protective effects against oxidative stress (34). Cellular response to oxidative stress is regulated at the transcriptional level through redox signaling pathways, with NRF2 playing a key role (33). Under normal homeostatic conditions, KEAP1 binds to NRF2 in the cytoplasm and results in ubiquitination and proteasomal degradation of NRF2. However, when tissues are subjected to ROS generated following exposure to atmospheric pressure plasma, excessive levels of oxidative stress inactivate KEAP1, allowing the accumulation and translocation of NRF2 to the nucleus (35, 36). In the nucleus, NRF2 binds to the antioxidant response element (ARE) on DNA and results in an antioxidant defense response by enhancing the expression of downstream antioxidant genes such as HMOX1 and NQO1 (37, 38). Thus, the controlled levels of ROS produced by CAP create an environment that closely mimics the natural wound healing process. Activation of NRF2 in diabetic mice leads to elevated TGF-β1 levels and reduced MMP-9 expression in the skin, accompanied by a decrease in oxidative stress. This reduction in oxidative burden, facilitated by NRF2 activation, enhances granulation tissue formation and matrix production, while also suppressing chronic inflammation and excessive MMP9 production (34). Additionally, exposure to cold plasma can enhance chronic wound healing by increasing NRF2 levels and promoting the expression of antioxidant and detoxification enzymes such as HMOX1 and NQO1 (39), GPX, CAT, SOD (37), thioredoxin, thioredoxin reductase 1, peroxiredoxin 1, HS90A, actin, and TCPA (40). These enzymes play crucial roles in neutralizing ROS and reducing oxidative stress within the wound microenvironment, mitigating the inflammatory response associated with excessive ROS production.

### Effects of cold atmospheric plasma on angiogenesis and proliferation

The proliferative stage of wound healing involves the coordinated interplay of different cell types, ECM components, and signaling molecules to rebuild and strengthen the damaged tissue. This key phase encompasses fibroblast migration into the wound, where they undergo morphological changes and secrete proteolytic enzymes for matrix penetration. Angiogenesis, driven by growth factors such as PDGF and TGF-*β*, ensures the formation of new capillaries. This phase is critical for the formation and accumulation of ECM components, establishing tissue strength and functionality. Additionally, angiogenesis, granulation tissue formation, and epithelialization facilitate wound regeneration. Impairment of the proliferative stage leads to delayed or incomplete wound healing (41). Cold plasma enhances cytokines, growth factors, and mediators, promoting angiogenesis, fibroblast and keratinocyte activity, NO production, and microcirculation (42, 43).

Compromised angiogenesis is a defining feature of chronic wounds in conditions such as diabetes (44). Cold plasma therapy has shown promising potential in promoting angiogenesis throughout the proliferative step of wound recovery through its ability to increase the expression of angiogenesis-related cytokines. Microwave-driven plasma torch increases angiogenesis-related molecules in endothelial cells, including proangiogenic factors (i.e., Angiopoietin-2 and Amphiregulin) and antiangiogenic factors (Angiostatin and Endostatin) (45) while increasing proangiogenic factors Artemin, Endothelin-1, IL-8, and Urokinase Plasminogen Activator (uPA) in keratinocytes, and uPA, TIMP-1, MMP-9, MCP-1, Endostatin, and Angiogenin in fibroblasts (45). Cold plasma treatment may promote vascularization by triggering interactions between endothelial cells, keratinocytes, and fibroblasts, affecting both pro- and anti-angiogenic factors. Endothelial cell proliferation is improved through increased expression of proangiogenic FGF-2 (46). However, the impact of cold plasma extends beyond direct mechanisms, with paracrine stimulation of various angiogenesis-related cytokines among endothelial cells, keratinocytes, and fibroblasts depending on cell-specific variations and sensitivity to CAP (45).

In addition to cytokines, plasma technology therapy induces the expression of various growth factors, significantly influencing angiogenesis and vascular remodeling. The TGF-*β* superfamily, including types 1, 2, and 3, serves as crucial regulators in wound healing, impacting cellular processes like proliferation, differentiation, migration, and survival (47). TGF-β1 and TGF-β2 play a dual role in wound healing; while influencing chemotaxis, collagen synthesis, angiogenesis, and reepithelialization in acute response, excessive levels of TGF-β1 and TGF-β2 are associated with scar formation. In contrast, TGF-β3, prevalent in early wound healing, acts as an on-and-off switch, controlling cell migration and promoting scar-free healing, ultimately reducing scar tissue development (48–50). In keratinocytes, treatment with the plasma torch system induces a significant upregulation of TGF-ß1 and TGF-ß2 on both mRNA and protein levels. Moreover, these TGF-ßs are notably increased in murine skin after repeated CAP treatments (51). The enhanced expression of TGF-ß1 and TGF-ß2 induced by cold plasma, along with their association with scar formation in the remodeling phase of wound healing, underscoring the need for caution in employing plasma therapy across different stages of wound healing. While plasma application during the proliferative stage increases the expression of key wound healing cytokines (TGF-ß1 and TGF-ß2), this very mechanism could potentially impede scar tissue resolution and result in excessive scarring.

During proliferation, activating molecular pathways is key for cell migration, growth, and differentiation in tissue repair. Treatment of MG63 cells with plasma technology promotes cellular proliferation during wound repair, as indicated by the upregulation of Ki67 and PCNA expression (52).

HIF-1α plays a crucial role in regulating angiogenesis, responding to hypoxic conditions by enhancing the production of angiogenic growth factors including VEGF and FGF. Inadequate responses to hypoxic stimuli can lead to chronic hypoxia, contributing to persistent wound formation, including pressure ulcers. Conversely, excessive HIF expression may result in heightened ECM deposition and fibrotic tissue formation (53). The precise regulation of HIF expression is critical for ensuring timely and effective wound healing while preventing the development of chronic wounds. In the context of plasma treatment, observed in keratinocytes, there is an increased expression of HIF and various angiogenic growth factors (Ang-1, Ang-2, VEGF-A, HB-EGF, PDGF-AA, PDGF-BB, FGF-2, and FGF-7) (54). Interestingly, in the smooth muscle cells of the pulmonary artery, ROS produced exogenously via H2O2 or through a NOX4-associated NADPH oxidase can trigger transcriptional activation of HIF-1α, which is a process reliant on NF-κB (55). The possible involvement of ROS in HIF-1α activation might act as the potential underlying mechanism in plasma-induced angiogenesis during wound healing, emphasizing the need for further investigations into this complex process.

Additionally, plasma-induced activation of endothelial NO synthase (eNOS) is observed in mouse models of burns, which contributes to acute wound healing by regulating cell motility, proliferation, and differentiation. Treating human microvascular endothelial cells with a plasma jet using helium gas leads to capillary tube formation, cellular migration, and acute wound closure, accompanied by increased expression of pro-angiogenic endothelial markers (42). Treatment with a DBD device using pure helium gas also influences the expression of galectin-1, −2, and − 3, which are involved in immune and inflammatory responses in wound healing. Galectin-1, in particular, regulates the neuropilin-1/Smad3/NOX4 pathway, myofibroblast formation, and ECM generation (56). In the proliferative phase of wound healing, TGF-*β*/Smad signal transduction pathway induces the transformation of fibroblasts into myofibroblasts, crucial for wound contraction (57). Additionally, CAP induces the expression of angiogenesis-related receptors FGF R1 and VEGF R1 in endothelial cells from human umbilical veins treated with cold plasma, suggesting autocrine mechanisms (45).

The HIPPO signaling pathway, responsible for regulating cell growth and tissue homeostasis, is stimulated by argon plasma jet (kINPen® MED) therapy. YAP, the nuclear effector of this pathway, is activated by plasma therapy, leading to paracrine signaling between dermal fibroblasts and keratinocytes. Plasma-induced YAP activation upregulates CTGF and Cyr61 also known as CCN1, which is a multifunctional regulator in wound healing, involved in angiogenesis, migration, and differentiation (58), and their expression is further enhanced when human keratinocyte cells are co-cultured with CAP-treated fibroblasts (59). It seems that generation of ROS from cold plasma therapy can induce communication between fibroblasts and keratinocytes through activation of the HIPPO pathway, possibly leading to ECM production and keratinocyte migration, resulting in subsequent wound healing.

Another important aspect of wound healing influenced by plasma therapy is the enhancement of microcirculation. Several studies have demonstrated the positive results of cold plasma application on microcirculation of the wound. Plasma-based treatment has been shown to enhance oxygen saturation and increase blood flow to the chronic wound, which can be particularly beneficial for diabetic non-healing wounds and improving cutaneous microcirculation parameters (60–63). Table 1 summarizes various studies examining the impact of plasma on wound healing processes and associated biological molecules.

**Table 1:** Overview of research investigating the impact of different plasma source types and gasses on wound healing through the modulation of biological molecules.

Plasma source type	Gas	Biological molecule	Type of molecule	Impact on wound healing	Reference
Microwave-driven plasma torch	Argon	Angiopoietin-2, Amphiregulin	Growth Factors	Enhances expression of proangiogenic factors	Arndt et al. (45)
Microwave-driven plasma torch	Argon	Angiostatin, Endostatin	Antiangiogenic Factors	Modulates anti-angiogenic factors	Arndt et al. (45)
Microwave-driven plasma torch	Argon	Artemin, Endothelin-1, IL-8, uPA	Cytokines	Indirect stimulation of keratinocytes and fibroblasts	Arndt et al. (45)
Microwave-driven plasma torch	Argon	TGF-β1, TGF-β2	Growth Factors	Upregulation impacts scar formation during healing	Arndt et al. (45)
Dielectric barrier discharge	Helium and air mix	HIF-1α	Transcription Factor	Regulates angiogenesis under hypoxic conditions	Cui et al. (54)
Dielectric barrier discharge	Helium and air mix	Ang-1, Ang-2, VEGF-A, HB-EGF	Growth Factors	Enhances expression of various angiogenic growth factors	Cui et al. (54)
Dielectric barrier discharge	Helium and air mix	PDGF-AA, PDGF-BB, FGF-2, FGF-7	Growth Factors	Enhances expression of various angiogenic growth factors	Cui et al. (54)
Plasma jet	Helium	eNOS	Enzyme	Promotes endothelial function and wound closure	Duchesne et al. (42)
Plasma jet	Helium	Endothelial markers	Cell Markers	Promotes capillary tube formation and cellular migration	Duchesne et al. (42)
Plasma jet	Argon	YAP	Signaling Protein	Activates HIPPO pathway, enhancing cell growth	Shome et al. (59) and Jun and Lau (58)
Plasma jet	Argon	CTGF, Cyr61 (CCN1)	Growth Factors	Influences ECM production and keratinocyte migration	Shome et al. (59) and Jun and Lau (58)

## Effects of cold atmospheric plasma on tissue remodeling

The remodeling stage in wound healing serves a dual role, aiming to both strengthen and refine the newly formed tissue (64). This phase involves the modification and reorganization of the ECM, primarily composed of collagen, to enhance tissue durability (65). Fibroblasts, key cellular players in this process, contribute significantly to tissue maturation by synthesizing and depositing collagen (66). Plasma therapy stimulates fibroblasts, resulting in actin cytoskeleton reorganization and enhanced collagen matrix production (67). Additionally, cold plasma enhances the expression of Collagen Type I *α* (COL1a), which is an important gene involved in collagen synthesis (52). Plasma-induced therapy also induces ECM remodeling by increasing the expression of the Matrix Metalloproteinase-1 (MMP1) gene (68). MMPs are crucial in both acute and chronic wounds through regulation of the balance between ECM degradation and deposition which is a vital process for wound re-epithelialization. Controlling MMP activity is crucial in chronic wounds to support tissue repair and wound closure (69). Additionally, MMP1 is crucial in wound recovery by facilitating keratinocyte migration on COL1 in injured skin as it cleaves collagen and allows keratinocytes to establish and release tight contacts with the dermal matrix, thus promoting re-epithelialization of the wound (68). A common outcome following remodeling in skin injuries is scar formation, which involves an excess of ECM and fibroblast activity. Abnormal scars develop when wound regeneration is disrupted by ongoing inflammation. The TGF-*β*/Smad cascade is recognized as the key regulator controlling collagen production in fibroblasts and myofibroblasts (70). CAP may hold potential in preventing scar development by suppressing TGF-β1, lowering α-SMA and COL1. In a study investigating the impact of cold helium plasma on scar development in acute wounds in rats, the non-thermal plasma-treated wounds exhibited accelerated healing from day 5 post-surgery, with significantly reduced scar width compared to the control group. Importantly, lower TGF-β1, p-Smad2, p-Smad3, α-SMA, and type I collagen were observed in cold plasma treated acute wounds (71).

### Antimicrobial effects

Plasma treatment demonstrates significant antimicrobial effects by reducing microbial load to levels comparable with those achieved by conventional biocides and antibiotics. Unlike traditional antimicrobial agents, plasma therapy does not promote the development of bacterial resistance, which is a major advantage (72, 73).

The bactericidal effects of CAP can be attributed to three primary mechanisms that disrupt bacterial cell structures and functions (74). Firstly, direct membrane disruption leads to the leakage of vital cellular elements including potassium, nucleic acids, and proteins, compromising the cell membrane’s integrity and leading to the release of crucial intracellular materials and contributing to cell death (75). The impact of cold plasma on bacterial structures varies depending on the type of bacteria, with Gram-negative bacteria experiencing substantial damage to their outer membrane, while Gram-positive bacteria show fewer morphological modifications (76). Additionally, membrane lipid peroxidation induced by ROS, such as singlet oxygen and hydrogen peroxide-like species, is identified as a major mechanism responsible for inactivation of bacteria, as shown when using the floating-electrode dielectric-barrier discharge (FE-DBD) technique in E.coli (77). Furthermore, plasma exposure depolarized the membrane in *S. aureus*, comparable to the effect induced by chlorhexidine treatment (72).

Secondly, CAP-generated reactive species damage microbial proteins, causing polymerization and functional loss, which destroys biofilms (78). CAP-induced oxidative damage to proteins, including SaFtsZ and SaClpP, collectively leads to *S. aureus* cell death within the biofilm. The short-lived species (•OH, O2, •NO, and ONOO−) were found to be crucial in modifying and inactivating proteins compared to the long-lived H2O2, NO2−, and NO3− (78).

The third mechanism involves direct chemical damage to DNA, where plasma-generated reactive species induce modifications and alterations to the genetic material. Plasma-induced DNA damage in bacteria results from the combined activity of reactive compounds (ROS and RNS), triggering proteins to interlink with DNA strands and leading to the formation of DNA-protein crosslinks that are challenging to repair (79).

Additionally, CAP enhances the expression of antimicrobial peptides, particularly defensins, which are positively charged tiny proteins functioning as antimicrobial peptides, in addition to modulation of immune cells and regulation of inflammatory processes (80). Cold plasma exposure was examined on the expression of human ß-defensins (HBDs). A 2-min treatment with a plasma torch system notably increased the levels of HBD-2 in keratinocytes, while HBD-1 and HBD-3 mRNA expression remained unaffected, suggesting that HBD-2 is the most important HBD influenced by CAP treatment (51). Additionally, the expression of mouse *β*-defensins (MBDs), specifically MBD-2 and MBD-3, was notably upregulated at the mRNA level in epidermal skin after five sessions of CAP therapy, whereas MBD-1 remained largely unchanged. Immunohistochemical analysis validated the elevated presence of MBD-2 in epidermal cells post-treatment with an argon plasma torch (51).

### Clinical applications of CAP

In 2010, the first randomized controlled trial (RCT) was conducted to assess the safety of CAP as a treatment for chronic wounds. This trial included 36 patients, accounting for a total of 38 wounds with chronic infections. The treatment regimen involved a daily 5-min exposure to cold atmospheric argon plasma, complementing standard wound care protocols. The study demonstrated a substantial 34% reduction in bacterial levels in the treated wounds, regardless of the bacterial strains. Importantly, the treatment was well-tolerated by patients, with no adverse side effects reported (81).

Following the success of non-thermal plasma in disinfecting the skin surface, further research was conducted to explore its effects on skin physiology and antioxidant profile. One study focused on evaluating the effects of CAP on the epidermal layer of human skin, known as the stratum corneum. The study involved seven healthy volunteers, and skin parameters including barrier function, hydration, temperature, and irritation were evaluated before and after cold plasma application. The results indicated a decrease in beta-carotene levels in the superficial stratum corneum post-cold plasma treatment, likely due to an increase in reactive oxygen species induced by plasma. Despite an observed increase in skin temperature, no damage to the skin or its functions was detected, suggesting the safety of cold plasma for clinical use (82).

Further studies supported the role of plasma medicine as a safe and effective method for reducing bacterial burden in non-healing wounds. In an RCT involving 24 patients with chronic infected wounds, a 2-min cold atmospheric argon plasma session was administered using two distinct plasma devices, in addition to standard wound care. Patients served as their own controls, with each patient’s wound assessed before and after treatment with both plasma devices. The study aimed to evaluate the safety and efficacy of these treatments in reducing bacterial load, irrespective of bacterial species or resistance levels. Results indicated a significant reduction in bacterial load following treatment with both devices. Importantly, no adverse effects were observed, highlighting the safety and tolerability of plasma treatment for chronic wounds (83). Further studies comparing the antibacterial effects of tissue-tolerable plasma (TTP) with existing methods yielded promising results. In one study, the efficacy of TTP was explored compared to a standard antiseptic solution (octenidine dihydrochloride with 2-phenoxyethanol) for reducing bacterial colonization on healthy human skin. TTP treatment demonstrated a significant reduction in bacterial load, consistent with previous studies reporting its efficacy against Gram-positive and Gram-negative bacteria, biofilm-producing bacteria, viruses, fungi, and spores. However, reductions in bacterial load were also observed with the standard solution, which exhibited slightly higher efficacy. Technical limitations, such as incomplete coverage of the treated area due to manual handling, may have contributed to the slightly lower effectiveness of TTP. Addressing these technical challenges through further research could enhance the efficacy of TTP. Nevertheless, TTP showed tissue tolerance, targeted efficacy on the skin surface and hair follicles, and absence of allergic reactions, demonstrating its potential as an alternative approach for managing skin diseases and wounds (84). The comparable antiseptic efficacy between TTP and ODC prompted interest in combining their antimicrobial effects for an enhanced treatment strategy in chronic wound treatment, and in the next study, 34 patients with chronic leg ulcers were divided into three treatment groups: TTP, ODC, and a combination of TTP followed by ODC. Treatments were limited to 1 cm^2^ of the wound surface, and TTP was applied using a plasma jet for 1 min. Wounds receiving combined TTP and antiseptic treatment initially had higher bacterial colonization compared to wounds in the other treatment arms. However, all antiseptic procedures led to a significant reduction in bacterial growth with the combined treatment group displaying more effective reduction of bacterial load compared to either treatment alone. The analysis of bacterial strains before and after treatments showed shifts in composition, with Staphylococcus and Pseudomonas being the most prevalent. The study also evaluated wound exudation and its relation to bacterial colonization, finding that strongly exuding wounds were more frequently colonized. Additionally, the evaluation of pain sensation associated with the antiseptic procedures revealed varying levels of discomfort for different treatment arms, with no significant differences detected (85).

A DBD device was evaluated for safety, and as secondary objectives, effectiveness and suitability as an additional therapy for chronic venous leg ulcers. Fourteen patients were randomly assigned to receive either standard wound care or plasma therapy in addition to standard care over an 8-week period. The application of plasma was generally well-tolerated, with only two patients reporting minimal discomfort. Moreover, it significantly reduced bacterial load and demonstrated potential for ulcer size reduction (86). In another study, a plasma source consisting of flexible plasma pads and a plasma driving unit was used on human skin sourced from healthy donors undergoing dermolipectomy or from deceased donors through a tissue bank. Safety assessments conducted on the dermal samples showed that viability was largely unaffected by plasma treatments, even over consecutive days. Additionally, the study found no significant increase in DNA damage due to plasma treatment. Patient’s reported transient warmth sensation and itching, considered acceptable levels of discomfort, and a temporary increase in skin temperature and redness was observed, which returned to baseline levels after 30 min. Notably, the plasma treatment exhibited antibacterial effects on the skin of healthy volunteers, with a significant reduction in bacterial count regardless of plasma power setting. These findings suggest promising applications for the flexible plasma device in promoting wound healing (87).

Recent trials show that CAP therapy significantly improves wound healing by reducing size, increasing granulation and vascularization, decreasing exudate, and lowering bacterial levels (summarized in Table 2).

**Table 2:** Overview of recent clinical studies on cold atmospheric plasma treatments for wound care.

Study Type	Year	Patients	Treatment	Main findings	Reference
RCT on diabetic foot ulcers	2020	43	CAP therapy vs. placebo	Notable enhancement in recovery, evidenced by reduced area and faster healing process, similar reduction in infection and microbial load, no therapy-related adverse events	Stratmann et al. (88)
RCT on helium plasma jet treatment	2020	44	Standard care alone vs. standard care + CAP treatment 3/week for three weeks	Plasma helium jet treatment reduced wound size, immediate antiseptic effects on bacterial load, not long-lasting	Mirpour et al. (89)
RCT on diabetic foot ulcers	2021	20	Standard management vs. CAP twice a week for six weeks in addition to standard care	Significant reduction in wound exudate after three weeks of treatment, improved wound grading by sixth week, reduced ulcer size by end of treatment period	Samsavar et al. (90)
Multicenter RCT on plasma jet therapy	2021	78	Plasma jet therapy vs. best practice wound dressings	Higher levels of granulation tissue, faster wound area reduction, more rapid decrease in wound pH value, swift overcoming of local infection with CAP-jet therapy, notable percentage of complete healing of chronic ulcers with CAP-jet therapy	Strohal et al. (91)
Pilot study on therapy-refractory chronic wounds	2022	37	Once-weekly vs. twice-weekly CAP treatment, placebo group	Significant decrease in wound area, observed pain reduction, bacterial load decreased, once-weekly CAP treatment equally effective as more frequent treatments	Moelleken et al. (92).
Pilot study on skin graft treatment	2021	10	CAP wound dressing vs. conventional therapy	CAP wound dressing outperformed control, well tolerated, lower sensation of pain	Van Welzen (93)
RCT assessing effectiveness of cold plasma in chronic diabetic wound	2022	23	CAP therapy vs. standard care	Enhanced granulation, vascularization, and re-epithelialization, increased expression of key growth factors and cytokines, significant reduction in wound area, observed pain reduction, enhanced quality of life related to wound, decreased bacterial load	Hiller et al. (94).
RCT on chronic venous leg ulcer	2025	46	Standard care vs. CAP in addition to Standard care	Higher wound healing rates in treatment groups compared to control (53.3% for once-a-week and 61.5% for twice-a-week treatment vs. 25.0% in control). Largest wound area reduction observed in twice-a-week group (95.2%). No serious device-related adverse events reported.	Bakker et al. (112)

In an RCT involving 43 patients, CAP notably accelerated diabetic wound recovery. The study found that plasma therapy led to a notable increase in chronic wound healing, as evidenced by a significant reduction in wound size and a faster time to achieve meaningful wound reduction. While reductions in infection and microbial load were similar between the plasma-based wound care and placebo groups, no therapy-related side effects were reported (88). In another randomized study on diabetic foot ulcers, 44 patients received standard care alone or in combination with plasma-generated treatment three times a week for three weeks. Plasma helium jet treatment effectively reduced chronic wound size, and a higher percentage of wounds in the standard care + CAP group reached a size of ≤0.5 compared to the standard care group. Additionally, cold plasma therapy demonstrated immediate antiseptic effects on bacterial load, although these effects were not long-lasting (89). In a subsequent clinical trial on diabetic foot ulcers, 20 patients were enrolled. The control group underwent standard practice treatment, while the other group underwent CAP-mediated wound care plus routine care. Patients receiving plasma care demonstrated significant decrease in exudate from wound, enhanced wound grading, and reduced wound surface upon completion of treatment (90).

In a comprehensive multicenter, randomized trial, researchers examined the effectiveness of plasma jet therapy against standard wound care practices (including rinsing, dressing, and debridement) in managing a diverse range of chronic wounds, both infected and non-infected, across 78 patients. The main outcome measures included total granulation tissue, decrease in wound size, treatment duration, alterations in acidity levels, infection severity score, exudate volume, and toleration. Plasma jet treatment increased granulation tissue, sped up wound healing, and reduced pH faster than control treatment. Additionally, plasma therapy led to quicker resolution of local infections. Notably, approximately 60%of patients receiving plasma jet therapy achieved satisfactory resolution of chronic ulcers within 6 weeks, in contrast to only 5%in the best practice group (91).

To assess the effectiveness of atmospheric pressure plasma on therapy-refractory chronic wounds a pilot study was conducted comparing once and twice weekly treatment intervals, as well as a placebo group. Both plasma therapy groups exhibited a significant decrease in wound area, with observed pain reduction. The group receiving plasma treatment twice weekly also showed additional improvement in wound-specific quality of life. Following 12 weeks of CAP treatment, bacterial load decreased by 50.4% for the once-weekly treated group and by 35.0% for the twice-weekly treated group, indicating that once-weekly cold plasma treatment is equally effective as more frequent treatments, providing a convenient and cost-effective option for clinical practice (92).

In addition to DBD and jet devices, a controlled preliminary investigation was conducted to assess the effectiveness and patient tolerance of a newly developed CAP dressing for acute wounds compared to conventional therapy in acute recovery skin grafts. The investigation involved 10 patients, each receiving a 7-day period of both treatments. Plasma-enhanced wound dressing was directly applied onto the distal half of the wound, while the proximal half underwent standard treatment with polyhexanide wound gel and fatty gauze. The plasma wound care surpassed the control in three critical wound parameters: deep tissue oxygen saturation, hemoglobin distribution, and tissue water distribution. Furthermore, plasma-based wound care exhibited excellent tolerability and reduced pain experienced in areas exposed to plasma (93).

In an RCT assessing the effectiveness of cold plasma therapy in addressing chronic diabetic foot ulcers, cytokine and growth factor expression from wound exudate samples were examined over a two-week treatment period. The findings emphasized the notable impact of plasma technology in enhancing granulation, vascularization, and re-epithelialization, with elevated levels of key GFs such as FGF-2 and VEGF-A, along with increased production of proinflammatory TNF *α*, IL-8, and IL-1α (94).

The diverse applications of atmospheric pressure plasma have demonstrated its potential as a safe and effective tool in wound healing, particularly in the treatment of chronic and therapy-refractory wounds, with promising results extending to diabetic foot ulcers. Moreover, CAP’s versatility extends beyond conventional wound healing, as it has shown promise as an innovative treatment approach for striae distensae (stretch marks) (95), in free flap procedures (96), enamel remineralization (97) and it may speed up recovery after oral surgeries (98).

Overall, cold plasma is a safe and effective treatment option for both chronic and acute wound healing, demonstrating notable reductions in bacterial load without adverse effects. Clinical trials indicate the efficacy of cold plasma in promoting wound healing, particularly in diabetic foot ulcers, where it accelerates the healing process and reduces wound size without significant adverse effects.

### Future trends and concerns in clinical application of cold atmospheric plasma

Atmospheric plasma therapy is a rapidly evolving area of research with promising potential for acute and chronic wound healing, but several issues need to be addressed in future studies for successful transition from bench to bedside. The main issues that need to be addressed include standardization of treatment protocols, exploration of combination therapies, development of portable devices, further understanding of the mechanisms behind cold plasma’s healing effects, assessment of tissue effects, evaluation of long-term safety, consideration of interaction with other therapies, and customization for different patient populations and wound types.

To establish standardized plasma medicine protocols, it is helpful to define specific parameters, such as the type of plasma device (DBD, APPJ, hybrid sources), optimal treatment duration, and voltage selection (99). The density and temperature of the plasma can vary based on the plasma generation source, the type of used gas, and the electrical power settings applied (21, 100). Establishing a unified protocol would allow a better understanding of plasma-induced healing potential in wound healing through facilitating comparisons across different studies. Moreover, it would allow researchers to explore the promise of combining plasma technology with other regenerative approaches such as growth factors and biomaterials for enhanced wound healing outcomes. In a pilot study, we showed that CAP has synergistic effects with amniotic membrane patches in enhancing wound healing, suggesting the potential to optimize their combined efficacy and improve clinical outcomes. However, given the diverse range of therapeutic approaches employed in wound management, understanding the potential interactions between ionized gas devices and other therapies is crucial. It is important to investigate the compatibility, synergistic effects, and potential conflicts between non-thermal plasma treatment and commonly used wound treatments, such as antibiotics, antiseptics, or topical medications, to minimize any potential adverse interactions (101).

Recent advances in plasma-based therapies have shown promise in a wide range of wound types, including intra-abdominal wounds. Plasma-activated media, previously shown to be effective in reducing cancer cell viability in breast and cervix cancer models (102), to prevent complications in surgical wounds, particularly within the peritoneal cavity (103). Human tissue-resident peritoneal macrophages exhibit resistance to oxidative stress from plasma-activated liquids, suggesting a potential immunomodulatory role for plasma therapies in preventing excessive inflammatory responses during intra-abdominal wound healing. Furthermore, plasma-activated media selectively inhibits pro-adhesive fibroblast activity while preserving the anti-adhesive function of mesothelial cells (104). This cell-specific anti-adhesive effect indicates that plasma-activated media could be a valuable tool in reducing postoperative adhesions, which remain a major cause of morbidity following abdominal surgeries. Incorporating plasma-based approaches into intra-abdominal wound management may reduce surgical complications and improve healing outcomes.

Essential for broader clinical applications, the development of portable and cost-effective plasma devices is underway. The creation of compact devices without compromising safety or efficacy facilitates low-temperature plasma use in diverse healthcare settings. Despite CAP’s demonstrated safety in wound healing (105, 106), assessing potential safety risks as investigations progress remains crucial. Future research should examine how non-thermal plasma affects healthy tissue to ensure it promotes healing without causing harm. Additionally, long-term safety profiles of plasma medicine need to be investigated to address any potential adverse effects or complications that may arise over extended periods.

As we explore the impact of plasma technology on healthy tissue and its long-term safety, it is important to turn our attention to the variability in its efficacy, which can be influenced by different patient populations and wound characteristics. Future research should explore cold plasma use across different patient groups and wound types, considering factors like age, comorbidities, and wound characteristics to improve outcomes. This will allow for the development of tailored treatment protocols to maximize the benefits of atmospheric plasma therapy in specific patient populations and optimize wound healing outcomes across diverse wound types.

## Conclusion

In this review, we provide an in-depth examination of the latest findings on the mechanisms, benefits, risks, and future directions of atmospheric pressure plasma application in acute and chronic wound management. Plasma therapy has promising potential in management of wounds due to its diverse influence on multiple cellular and molecular processes involved in tissue repair, including modulation of inflammation, promotion of angiogenesis, acceleration of tissue remodeling, and antimicrobial effects.

Safe use of plasma therapy in wound healing requires recognizing its risks and limitations. This involves not only standardizing non-thermal plasma devices and treatment protocols but also conducting comprehensive safety assessments. Previous preliminary studies (107, 108) have indicated no significant side effects with plasma medicine and demonstrated its efficacy in reducing microbial load without adverse effects on treated wounds (109–111). However, it is important to address safety concerns, such as the potential for tissue damage and adverse effects on surrounding healthy tissue, through comprehensive evaluation and future research efforts. Establishing robust safety measures will contribute to the responsible implementation of cold plasma interventions in clinical practice.

**Figure 3:**
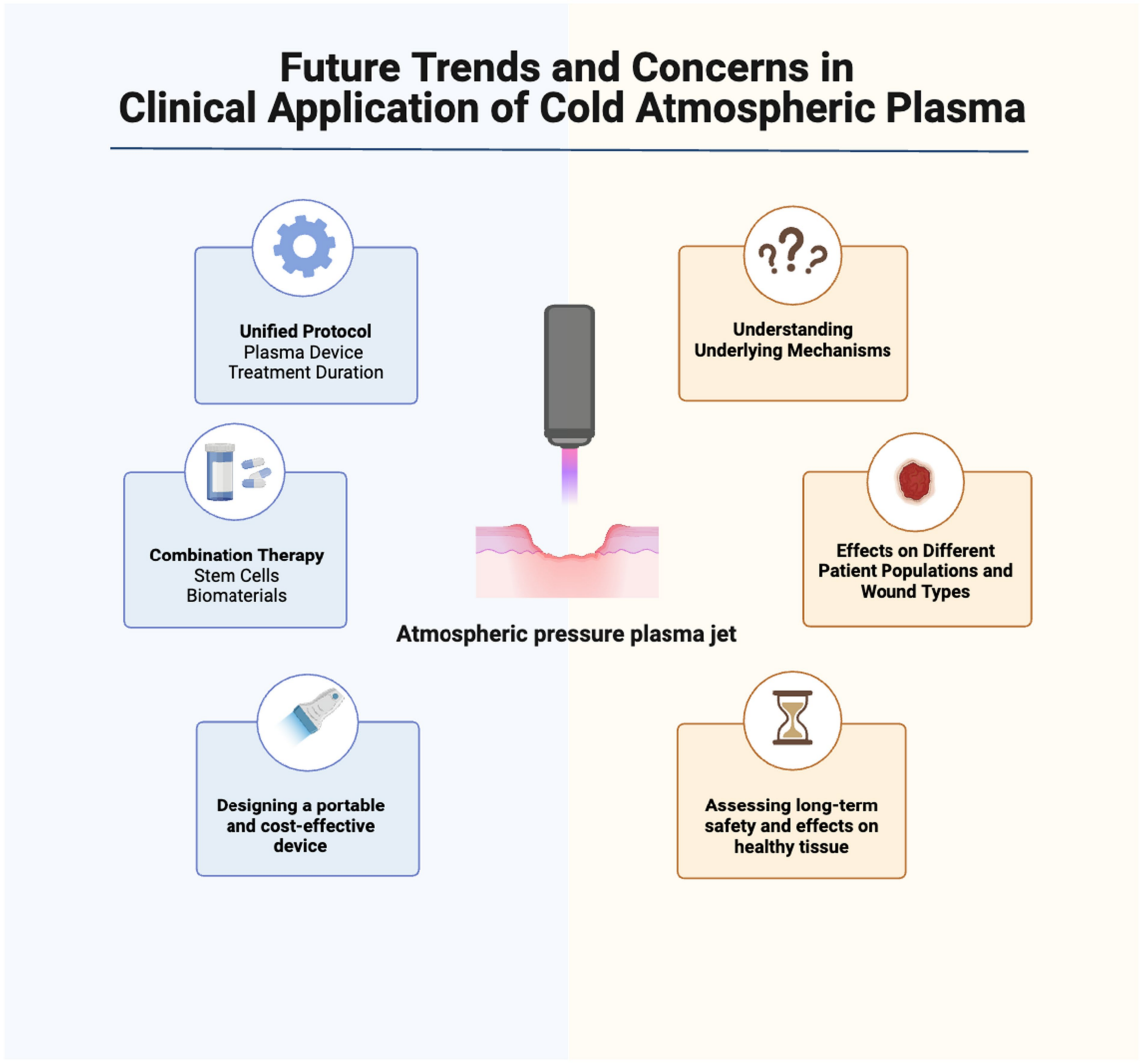
Future trends and concerns in clinical application of cold atmospheric plasma for wound healing. Further studies are needed to understand CAP’s interactions with cellular/molecular processes. Safety concerns, optimization of plasma application parameters, and use with other therapies must be evaluated. Cost-effectiveness and scalability of non-thermal plasma treatments, including affordable/portable devices, may be future research areas. Created with Biorender.com.

Future directions in low-temperature plasma research for wound healing are promising, with ongoing studies focusing on optimizing treatment parameters, elucidating the underlying mechanisms, and exploring novel applications, such as combination therapies with other wound healing modalities. Further research can clarify long-term effects and set protocols (Figure 3).
